# Compliance of Parenting Magazines Advertisements with American Academy of Pediatrics Recommendations

**DOI:** 10.3390/children3040023

**Published:** 2016-11-01

**Authors:** Michael B. Pitt, Jennifer N. Berger, Karen M. Sheehan

**Affiliations:** 1 Department of Pediatrics, University of Minnesota Masonic Children’s Hospital, M653 2450 Riverside Avenue South, Minneapolis, MN 55454, USA; 2 Ann & Robert H. Lurie Children’s Hospital of Chicago; 225 E Chicago Avenue, Chicago, IL 60611, USA; berge606@umn.edu; 3 Northwestern University’s Feinberg School of Medicine, 420 East Superior Street, Chicago, IL 60611, USA; ksheehan@luriehcildrens.org

**Keywords:** advertising, parenting magazines, American Academy of Pediatrics recommendations

## Abstract

This study examined 3218 advertisements from the two parenting magazines with highest circulation in the United States. The authors compared each advertisement for a product for use by children, against all the published recommendations of the American Academy of Pediatrics (AAP) on topics such as toy safety, helmet use, age-defined choking hazards, infant sleep safety, and others. Any advertisement with images or products which went against a published AAP recommendation was deemed as non-adherence and was categorized according to the statement it contradicted. Nearly one in six (15.7%) of the advertisements contained example(s) of non-adherence to AAP recommendations, with twelve categories of offense represented. Categories ranked by overall share from most to least include: non-Food and Drug Administration (FDA) approved medical treatments, age-defined choking hazards, vitamins, cold medicine, formula, oral care, screen time, toy/playground safety, infant sleep, nutrition, water safety, and fall risk. Given that repeated exposure to messages in advertisements has been associated with changes in health decision-making, and parents often turn to parenting magazines for advice and ideas regarding their children, the publishers might consider screening the content in order to prevent confusing and potentially dangerous messages from being disseminated in the media.

## 1. Introduction

Magazines remain an influential source of health information for women and mothers and have been shown to influence decisions about their own health and their children’s [1,2,3]. Messages about health are not only found in the editorial content of the magazine, but are often present in the advertisements. These messages may be explicitly stated in the product being advertised (i.e., “Take this pill to feel better”) or they may be implied by the images used in the advertisements. For example, an advertisement for lemonade which shows a child riding a bicycle without a helmet may subtly imply this behavior is safe. Whether stated or unstated, the messages provided in magazine advertisements may act as repetitive visual reinforcers, normalizing behavior. When these messages are inconsistent with public health recommendations, they may inadvertently lead to unsafe practices. 

Consumers often have the false assumption that if a product is being sold or advertised, it is safe to use [4]. Our fear is that seeing these potentially dangerous messages in advertisements within a magazine the readers trust may give the false impression that the product or behavior advertised is safe even if the advertised product itself is deemed dangerous by public health officials. While less clear, it is possible that the repeated use of imagery depicting unsafe actions may normalize this behavior. Simply seeing the images repeatedly of infants sleeping prone, for example, may add to the confusion created by inconsistencies between the media and the public health initiatives regarding safe sleep practices [4,5]. Others have shown that repetitive exposure to images and messages in advertising can alter decisions affecting health. Foss and Southwell showed that when they reviewed 28 years of *Parents Magazine* content for advertisements with images of infant feeding, there was generally decreased proportion of mothers who breastfed the next year after an increase in advertisements depicting bottle feeding [6]. Sobel et al. found that mothers who could recall specific advertisements for formula were twice as likely to use formula over breastfeeding [7]. Willinger et al. showed that the media strongly influenced caregivers choice of infant sleeping position [5]. Similar changes in behavior in response exposure to advertisements have recently been reinforced with exposure to alcohol [8] and cigarette advertising [9], both increasing subsequent use, as well as exposure to advertising of food contributing to childhood obesity [10]. 

The American Academy of Pediatrics (AAP) issues consensus statements and guidelines on many important issues facing children’s health and safety. Others have reported the prevalence of messages in magazine advertisements being contradictory to specific AAP recommendations including promoting vitamin use in children [11], excess fluoride intake [12], inadequate sun protection [13], and unsafe infant sleep positions [4]. Our objective was to expand on these analyses and determine the frequency and categories of advertisements for products for children in the top parenting magazines which contradict all published AAP recommendations, rather than a single category. Of note, it is not our intent to endorse any of these recommendations, some of which may be unrealistic, but rather to use them as a standard for public health recommendations for children at the time of publication. 

## 2. Methods

We obtained magazine readership data for the calendar years 2009 and 2014 from the Alliance for Audited Media. We chose the magazines in the parenthood category which had more than one million in total circulation in both years studied: *Parents* (2.21 million in 2009; 2.13 million in 2014) and *FamilyFun* (2.20 million in 2009; 2.13 million in 2014) [14,15] (Note *Parenting* magazine had 1.2 million in paid circulation in 2009, but was bought by *Parents* and ceased publication in 2013 and was therefore not included as comparisons could not be made).

As we were most interested in the messages found in advertisements for children’s products, we analyzed every advertisement during the study years to determine which products were intended for children’s use (e.g., toys, bedding, car seats, etc.) or consumption (e.g., food, non-alcoholic beverages, formula, children’s vitamins, etc.). We excluded any advertisement if it was stated or clearly implied that the product was intended for adults or pets (e.g., adult vitamins, beauty supplies, pet food, housewares, single food ingredients intended for the food preparer like vinegar or flour, etc.). Services that were clearly intended for the adult decision-maker were also excluded even if the child may obtain some benefit from the service (e.g., health insurance, hotel chains, photo studios, chamber of commerce advertisements, etc.). 

Each unique advertisement for a product was counted as one advertisement. Consecutive multi-page spreads advertising the same product were counted once as they represented a single advertisement for that product albeit spread over more than one consecutive page. Advertisements for a single retail outlet with multiple different products in the image (e.g., an advertisement for a grocery chain with a cart full of different products) were also counted as one advertisement as the retail outlet was the primary product advertised. Advertisements which promoted multiple unique products each available from individual vendors which were listed separately in the advertisement were counted with each individual product as its own advertisement. 

We reviewed all the AAP Policy Statements and *Where We Stand Statements* and used their parenting website https://healthychildren.org to compile a list of AAP recommendations that were current in the years studied. We then assessed whether or not the product or any images in the included advertisements had any inconsistencies with these recommendations. Some recommendations changed between 2009 and 2014; as we aimed to determine if products or images used in advertisements contradicted public health messages from the AAP at the time the advertisement ran, the advertisements were coded for contradictions that were current as of the calendar year of publication. If an advertisement contained more than one category of offense (for example an advertisement depicting an infant sleeping on his stomach while his sister walked by using an infant walker) each offense was counted once and assigned its specific category. We calculated total number of offenses for each year and determined both the percent of advertisements with at least one offense, and the breakdown by percent share of offenses by category (e.g., unsafe sleep, toy safety). 

We chose to evaluate two non-consecutive years to assess for consistency of categorization of offenses. We compared the proportion of advertisements with inconsistencies to AAP recommendations across the two years overall and by category of offense using Fischer’s exact test. Statistical significance was determined at the 0.05 level. No adjustments were made for multiple comparisons. SAS V9.3 (SAS Institute Inc., Cary, NC, USA) was used for the analysis. We assessed interrater reliability using Cohen’s Kappa statistic with one author reviewing each issue for the calendar year, and a second author assessing two issues as a sample from each year ensuring that more than 10% of advertisements reviewed were coded by each author. In advertisements which depicted children in possible violation of a recommendation with age-specific criteria (e.g., choking hazards by age) both reviewers (each pediatricians) needed to agree on the likely age of the child depicted for it to be included.

## 3. Results

There were 44 issues circulated during the study period (10 per year of *FamilyFun*; 12 per year of *Parents*). Kappa reliability between the two coders for ad violations, with each reviewing a shared 10.7% of the sample, ranged from 0.91 to 1.0, indicating excellent interrater agreement. Overall, 3218 advertisements were reviewed (1845 in 2009; 1373 in 2014) of which 2047 (63.6%) met the inclusion criteria. Of these, 321 advertisements (15.7%) contained one or more inconsistencies to AAP recommendations, with 396 violations in total (Table 1). On average, *FamilyFun* contained 5.0 offending advertisements per issue and *Parents* had 9.3 per issue. We identified twelve categories of offense type. The categories representing the type of offense are depicted in Table 2 with examples of advertisements from each category provided in Table 3. 

There was no significant difference in the total percentage of non-adherence between 2009 and 2014 (16.7% vs. 13.5%; *p* = 0.07). Several violation categories showed significant (*p* < 0.05) changes over the five years including decreases in percentages in the categories of oral care, sleep safety, and screen time, and increases in percentages of offenses in the non-Food and Drug Administration (FDA) approved medical management and fall risk categories.

The categories of non-adherence are described in detail below, listed in descending order of frequency over both years, grouped first by those offenses which may be life-threatening.

### 3.1. Potentially Life-Threatening Categories 

#### 3.1.1. Non-FDA Approved Therapy/Potentially Harmful Medical Mismanagement (*n* = 64 over both Years; 16.2% of Offenses)

While the AAP acknowledges that many families turn to natural over-the-counter remedies when their child is ill, they warn that they can be unsafe as they are not regulated by the FDA for purity, potency, efficacy or safety. They recommend not using such therapies without first consulting with the child’s pediatrician [16,17]. Advertisements were considered inconsistent with this recommendation if they were for a non-FDA approved over-the-counter supplement or device being marketed as a medical remedy or diagnostic tool for a potentially harmful condition. Vitamins were addressed in a separate category.

Examples of products advertised to treat potentially harmful conditions included supplements to treat abdominal pain, sinusitis, ear infections, acute conjunctivitis as well as supplements to treat childhood depression, anxiety, low confidence and daydreaming. An example of an advertisement which encouraged self-diagnosis was for a middle ear monitor which reportedly beeps in the presence of acute otitis media. 

#### 3.1.2. Choking Hazards (*n* = 59; 14.9%)

Choking is a leading cause of injury and mortality in children with nearly two-thirds of choking deaths occurring in infants and toddlers. As such, the AAP has age-specific choking recommendations minimize exposure to choking hazards (i.e., no peanuts, popcorn, hard or gummy candy under age four; no latex balloons under age eight) [18,19]. The second largest share of advertisements which contained messages inconsistent with AAP guidelines were for those regarding these age-specific choking hazards. The majority of the advertisements which violated choking guidelines were for gummy vitamins (*n* = 50; 84.7%) which the advertisers indicated as intended for ages two and up. The shape and consistency of these vitamins is the same as gummy candy which the AAP does not recommend children consume until at least age four [18]. Several advertisements also depicted young children with latex balloons, which the AAP recommends avoiding until age eight, as they contribute to 29% of choking deaths [18].

#### 3.1.3. Cold Medicine (*n* = 49; 12.4%)

The AAP recommends that children under six years old should not be given cold medicines citing lack of proven efficacy and health risks [20,21]. Despite this recommendation, 12.4% of the offending advertisements were for cold medicine promoted for use under the age of six.

#### 3.1.4. Unsafe Toys/Playground Safety (*n* = 21; 5.3%)

Injury is the leading cause of death in children in the United States [22]. Many unintended injuries in children occur in the context of play. Accordingly, the AAP has several guidelines for safe play including: avoiding infant walkers which have been shown to lead to falls and broken bones [23]; toys with small magnetic parts which if swallowed can link up leading to intestinal obstruction [24]; backyard trampolines which are associated with injuries [25,26]; non-powder projectile guns [27]; helmet use [28]; and playground safety [29]. There were examples of non-adherence for all of these categories, with advertisements for infant walkers making up the largest proportion (*n* = 9; 43% of this category). Injuries from infant walkers lead to thousands of emergency department visits each year leading the AAP to call for a ban on their sale in the United States, mirroring those present in other countries [23].

#### 3.1.5. Sleep Safety (*n* = 20; 5.1%)

Sudden Infant Death Syndrome (SIDS) is the fourth leading cause of death in infants [22]. Unsafe sleep positions and environments increase the risk for SIDS [30]. Others have reported that the majority of images in magazines depict sleeping infants in unsafe positions or environments [4]. In our study, advertisements were deemed inconsistent with the AAP’s recommendations on safe infant sleep position if they showed an infant sleeping on his or her side or stomach, or depicted an infant in an unsafe sleeping environment such as a nap cushion, or in a crib with bumpers, toys, or pillows [30]. This category made up 5.1% of the overall offenses.

#### 3.1.6. Pool/Water Safety (*n* = 12; 3.0%)

The AAP provides recommendations on the prevention of drowning, which is a leading cause of death in children [31,32]. Examples of advertisements found inconsistent with these recommendations included those showing children jumping off high rocks into lakes or on boats without life jackets. In 2009, advertisements for products which promoted swimming lessons for children under the age of four were considered non-adherence, as this was a contradiction of the AAP recommendation at that time. Their recommendations around swimming lessons changed in 2010, and as such, advertisement for infant swimming lessons were not considered offenses in 2014 [32]. 

#### 3.1.7. Fall Risk (*n* = 7; 1.8%)

The AAP has recommendations on shopping cart and high chair safety regarding fall risk as both are significant causes of injury leading to tens of thousands of emergency department visits in the United States each year [33,34]. Advertisements were deemed inconsistent with this recommendation if they showed a child in the main grocery basket of the shopping cart, or clearly not strapped in a high chair.

### 3.2. Non-Life Threatening Categories 

#### 3.2.1. Vitamins/Supplements (*n* = 53; 13.4%)

Vitamins and mineral supplementation may pose significant health risks [35,36]. The AAP states that healthy children receiving a normal, well-balanced diet do not need vitamin supplementation [17,37]. As such, advertisements for multivitamins for children, other than appropriately dosed Vitamin D monotherapy which the AAP recommends, were included as inconsistent with AAP recommendations and comprised 13.4% of the offenses. 

#### 3.2.2. Infant Formula (*n* = 44; 11.1%)

In order to encourage mothers to adhere to its recommendation for exclusive breastfeeding, the AAP joins the World Health Organization with a policy against direct-to-consumer advertising of formula [38,39]. As such, advertisements for infant formula were deemed inconsistent with this recommendation and made up 11.1% of offenses.

#### 3.2.3. Oral Care (*n* = 27; 6.8%)

Excess fluoride intake can lead to permanent staining of the teeth [40]. Several advertisements in the 2009 issues were for fluoridated toothpaste promoted under the age of two years which was inconsistent with AAP recommendation at that time and thus counted as non-adherence during that year. In 2014, the AAP changed its recommendation that children under the age of two years not use fluoridated toothpaste, to recommending its use in small amounts at the onset of tooth eruption. As such, advertisements promoting fluoride use for children under two were not counted as violations in the 2014 issue and as a result, there was only one oral care violation in that year. This advertisements was for teething gel for an infant, which is inconsistent with the AAPs teething recommendations, as these gels can pose risks including local reactions, numbing of the back of the throat which may lead to difficulty swallowing, seizures with overdose, and methemoglobinemia [41,42].

#### 3.2.4. Screen Time (*n* = 23; 5.8%)

The AAP recommends that children under age two not watch any television or videos on any device citing concerns about viewing interfering with normal development [43]. Advertisements for videos, television shows, or apps were deemed inconsistent with this recommendation if they explicitly stated in the advertisement that the program was recommended for viewing under the age of two. Advertisements which depicted children less than two using a smart device or watching television were also deemed as inconsistent with this recommendation.

#### 3.2.5. Nutrition (*n* = 17; 4.3%) 

While many of the advertisements were for food which may be considered unhealthy, the AAP does not explicitly recommend against anything in moderation with the exception of its recommendation to avoid fruit juice in infants [44]. As such we did not include food advertisements as non-adherence even if the food would be considered unhealthy. 

Advertisements for supplemental toddler formula were deemed inconsistent with the AAP’s recommendations for introduction of cow’s milk at the age of one year, as doing so earlier can lead to intestinal bleeding and inadequate absorption of nutrients [45]. In 2011, the AAP issued a statement advising against children drinking sports or energy drinks. There were three advertisements in 2014 for such drinks [46].

## 4. Discussion

Over the course of two years’ worth of issues separated in time (2009 and 2014), the advertisements for products intended for children in the two leading parenting magazines promote messages that are inconsistent with specific AAP recommendations on nearly one out of six occasions (15.7%). Of these inconsistencies, more than half (58.6%) were for advertisements with potentially life-threatening messages. 

Repeated exposure to messages in advertising has been shown to affect behavior and healthcare decision-making including the choice to formula feed [6,7], consume alcohol [8], initiate smoking [9], and make decisions about what we eat [10]. While these behavior changes were found following exposure to intentional advertising for products (formula, alcohol, etc.), less is known about the impact on behavior of the imagery used when unrelated to the product itself (e.g., an advertisement for sunscreen showing a toddler on a boat not wearing a life jacket). Further study is needed to determine if this more passive consumption of messages affects behavior. 

While parents are certain to receive messages about child rearing outside of parenting magazines (i.e., online, in parenting books, from trusted sources) we chose this media as a finite resource with the ability to easily make comparisons over time. We also found that certain groups, such as low-income pregnant women, report turning to magazines for information about their pregnancies more than the internet [1].

We acknowledge that some of the recommendations of the AAP may seem too strict or unrealistic. We were not looking, however, to validate or endorse the statements of the AAP, but rather to assess adherence to the current published statements in the messages of advertisers. Specifically we acknowledge that the AAP’s stance on children’s vitamins may seem inconsistent with societal norms as nearly one in three children in the United States reportedly take vitamins or supplements [47]. It is worth noting, however, that all but three of the advertisements for vitamins were gummy vitamins indicated as being safe for starting at age two, and therefore would have counted as non-adherence under the choking risk criteria regardless. As such, inclusion of this category does little to change the overall contribution to violations of AAP recommendations. 

Many have shown that frequent exposure to advertising for formula may contribute to decreased initiation or duration of breastfeeding [7,48,49]. Yet including formula advertisements may seem by some as too strict as many mothers choose to use formula for reasons ranging from convenience to inability to produce milk. However, these advertisements are in direct contradiction to the AAP’s published stance against advertising formula directly to mothers which aligns with the World Health Organizations call to ban advertising of breast milk substitutes [50]. If we remove this category, there were still 281 advertisements with non-adherence to AAP recommendations (13.7% of all advertisements). 

The choice to compare non-consecutive years of the magazines for categories of non-adherence was to determine if the proportion of offenses and their categorizations held up over time, and to ensure that they weren’t disproportionately weighted by a handful of advertising campaigns with offenses running repeatedly in that year. While the overall difference in percentage of non-adherence from 2009 to 2014 did not reach statistical significance, there were several categories with statistically significant changes. Some of these changes can likely be attributed to the inclusion criteria changing because of changes in AAP recommendations. The near elimination of non-adherence in the oral care category, for example, is most certainly due to the change in the AAP’s recommendation on fluoride use after 2009 which eliminated the violations which showed toothpaste marketed under the age of two. We did not, however, assess the volume of toothpaste depicted on a toothbrush in our analysis, and others have shown that over 96% of toothpaste advertisements in parenting magazines depict a toothbrush with a full swirl of toothpaste which is four times the recommended amount for children [12].

There were statistically significant decreases in non-adherence in the screen time and sleep safety categories. Both have received considerable media and advocacy attention in the last several years which may have influenced advertisers in the imagery used. In fact, there was only one advertisement in 2014 which depicted an infant in an unsafe sleep position compared to seventeen in 2009. When comparing to the 2007 study of 28 magazines for women which showed two-thirds of the infants in unsafe sleep environments, one must note that that study looked at all images in magazines, whereas ours only included advertisements for children [4]. Recently, Goodstein et al. evaluated 26 magazines and stock photo websites in 2014 looking for how infants were depicted while asleep; they found 61% of the unique magazine images depicted infants in unsafe positions, a similar percentage to the 2007 study [51]. Regardless, the fact that the absolute number of sleep violations in the advertisements reviewed dropped seventeen-fold over five years is promising. 

The increase in the fall risk category is likely because there were two advertisements in 2014, which each ran more than once, making up the seven offenses in this category that year. This underscores the importance at looking at the overall breakdown across the two years for a more meaningful cross section of the types of non-adherence seen. It is unclear why there were increases in the non-FDA approved medical management category, but the near doubling of this offense does merit future study. 

Our study has several limitations. We did not categorize the excluded advertisements, and therefore are not able to report the offending advertisements in proportion to their specific categories (i.e., that percentage of cold medicine advertised in these magazines indicated they were for use under the age of six). We chose to include only the advertisements which were for products for use by children as we were looking at pediatric recommendations. As a result, we do not report the advertisements for adult products which may still have had an image which contradicted an AAP recommendation (e.g., an advertisement for a detergent depicting an infant sleeping on her stomach). As others have reported these advertisements may also include contradictory messages, future studies should consider including these as well. There may be limitations to the generalizability of the study as we coded only two magazines and did the coding ourselves through the lens of researchers looking for violations. However, the fact that the two magazines studied have a combined circulation greater than all of the other magazines in the parenting category combined [8,9] makes the results reflective of the vast majority of the advertisements seen by the magazine readers in this category. While our coding was completed by two authors in the study, it was done with an a priori list of potential violations sourced from the AAP recommendations, and while it is possible some of these contraindications might not be noticed by the average reader, we were attempting to look purely at whether contraindications existed or not. Further study is needed to determine which types of messages are noticed and/or acted upon by readers. 

We feel magazine publishers should consider the products advertised in their issues and the messages they may send to their readers who are turning to them for parenting advice. Most of the offending advertisements (74%) were counted as offenses because of the actual product itself contradicting AAP recommendations such as infant sleep cushions, infant walkers, benzocaine containing teething gels, etc., many of which the publishers could consider prohibiting to promote agreed upon safety best practices. While it is unlikely they might consider barring advertisements for more mainstream, widely accepted products such as formula or vitamins, it is worth noting that none of the formula advertisements over the two years were in *FamilyFun*. While unclear if this omission was intentional, it indicates it is possible to avoid certain advertising content. Some of the offending advertisements were included not because of the product, but because of the background imagery which depicted unsafe practices. It is quite possible that the advertisers are not knowingly choosing to portray behavior that is unsafe for children and might be open to change in future campaigns if made aware. As such, it would be possible for magazine publishers to provide a list of images for advertisers to avoid (e.g., infants sleeping on their sides or stomachs, cribs with bumpers, toddlers holding latex balloons, children on boats without life jackets or bikes without helmets, etc.) without risking turning down advertisers whose products couldn’t be featured. 

## 5. Conclusions

The advertisements for products for children in the leading parenting magazines contained non-adherence to explicit AAP recommendations on nearly one out of six occasions (15.7%), over half of which contained messages which may be life-threatening. As these magazines are where many parents turn for advice, these messages may lead to reinforcing unsafe behavior putting children at risk. Magazine publishers could consider having advertising policies which provide guidelines on the content of the advertisements (products and images) they allow, in order to prevent confusing and potentially dangerous messages from being disseminated in the media. 

## Figures and Tables

**Table 1:** Proportion of advertisements with non-adherence for American Academy of Pediatrics (AAP) recommendations in the top two parenting magazines.

	2009	2014	*p* Value *	Total
*Parents*	140/774 (18.1%) [15.4%–21.0%]	82/540 (15.2%) [12.3%–18.5%]	0.18	222/1314 (16.7%)
*FamilyFun*	65/439 (14.8%) [11.6%–18.5%]	34/294 (11.6%) [8.1%–15.8%]	0.23	99/733 (13.5%)
Total	205/1213 (16.9%) [14.8%–19.1%]	116/834 (13.9%) [11.6%–16.4%]	0.07	321/2047 (15.7%)

* Fischer’s exact test: statistical significance was determined at the 0.05 level (95% confidence interval of the proportion).

**Table 2:** Comparison of categories of non-adherence of AAP recommendations in advertisements in top two parenting magazines over five years.

Violation Category	Overall *n* = 396 Violations % (*n*)	2009 *n* = 265 Violations % (*n*)	2014 *n* = 131 Violations % (*n*)	Change ↑↑↑ or ↓↓↓ Indicate Statistical Significance	*p-*Value *
**Non-FDA Approved Therapy/Self-Diagnostics**	16.2% (64)	11.7% (31)	25.2% (33)	↑↑↑	<0.001
**Choking Hazards**	14.9% (59)	16.2% (43)	12.2% (16)	↓	0.37
Vitamins (besides vit. D)	13.4% (53)	14.0% (37)	12.2% (16)	↓	0.75
**Cold Medication (<age 6)**	12.4% (49)	11.3% (30)	14.5% (19)	↑	0.42
Formula	11.1% (44)	9.1% (24)	15.3% (20)	↑	0.09
Oral Care ^±^	6.8% (27)	9.8% (26)	0.8% (1)	↓↓↓	<0.001
Screen Time (<age 2)	5.8% (23)	7.5% (20)	2.3% (3)	↓↓↓	0.04
**Toys/Playground Safety**	5.3% (21)	5.7% (15)	4.6% (6)	↓	0.81
**Sleep Safety**	5.1% (20)	7.2% (19)	0.8% (1)	↓↓↓	<0.01
Nutrition (sports drinks/toddler supplemental formula)	4.3% (17)	4.2% (11)	4.6% (6)	↑	0.80
**Pool/Water Safety ^±^**	3.0% (12)	3.4% (9)	2.3% (3)	↓	0.76
**Fall Risk**	1.8% (7)	0% (0)	5.3% (7)	↑↑↑	<0.001

**Bolded categories** represent violations which could be life-threatening (58.6% overall). * Fischer’s exact test: statistical significance was determined at the 0.05 level; ^±^ Indicates a change in the inclusion criteria based on a loosening of the recommendation between 2009 and 2014. FDA: Food and Drug Administration.

**Table 3:** Examples of advertisements/products depicting non-adherence for each category of AAP recommendation.

**Non-FDA Approved Therapy/Self-Diagnostics** A flower-based supplement to lower anxiety, decrease fear of school, nightmares, and tantrums as well as one to increase self-confidence and decrease daydreaming Homeopathic products for teething, ear aches, pink eye, sinusitis A device to place in the ear which beeps if there is an ear infection	**Choking Hazards (Age-Specific)** A popcorn seasoning with a picture of toddlers eating popcorn (AAP recommendation > 4 years) Gummy vitamins advertised for use under the age of 2 (AAP recommendation >4 years for gummy candy) Party decorations with pictures of toddlers holding latex balloons (AAP recommendation >8 years)	**Cold Medication** Cough medication promoted for use starting at the age of 2 (AAP recommendation > 6 years)
**Sleep Safety** An advertisement for formula promoting better sleep which showing an infant sleeping on his side Sleep cushions/bumpers for infants	**Toy/Playground Safety** Infants/toddlers using walkers Backyard trampolines Advertisement with pictures of kids using motorized personal vehicles without helmets Small magnetic beads	**Pool/Water Safety** Kids holding hands jumping off a cliff into water Pictures of children boating or waterskiing without life jackets 2009 Only: Advertisements for swim lessons for children under 4 years (recommendation against infant swim lessons changed in 2010)
**Fall Safety** Toddler standing in the grocery basket of a shopping cart being pushed by another child Young toddler in a booster seat balanced on a kitchen chair (not strapped)	**Vitamins (non-Vitamin D monotherapy)** Multivitamins (almost all gummy vitamins) targeted for children Mega-doses of vitamins in marked excess of recommended daily allowances (e.g., B12 supplements)	**Infant Formula** Any advertisement for infant formula was included Some claimed proven benefits of benefits for brain, growth, and eye development
**Oral Care** Benzocaine containing teething gels 2009 Only: Advertisements for fluoride toothpaste recommended under the age of 2 (recommendation was changed in 2014, and thus not counted as a violation in that year)	**Screen Time (under the age of 2)** DVDs promoted to spark baby’s learning and development DVDs to promote babies learning to read with a picture of an infant in a graduation cap Advertisements with infants using tablet computers	**Nutrition** A supplemental toddler formula with the tagline to give her this formula “instead of milk” Advertisements for sports drinks for children

Gray squares indicate categories that are potentially life-threatening.
